# Dual controls of vapour pressure deficit and soil moisture on photosynthesis in a restored temperate bog

**DOI:** 10.1016/j.scitotenv.2024.178366

**Published:** 2025-02-01

**Authors:** Sandeep Thayamkottu, Mohit Masta, June Skeeter, Jaan Pärn, Sara H. Knox, T. Luke Smallman, Ülo Mander

**Affiliations:** a Institute of Ecology and Earth Sciences, University of Tartu, Vanemuise Street. 46, 51003 Tartu, Estonia; b Department of Geography, McGill University, Montreal, QC, Canada; c Department of Geography, The University of British Columbia, Vancouver, BC, Canada; d School of GeoSciences, The University of Edinburgh, Edinburgh EH9 3FF, United Kingdom; e National Centre for Earth Observation, The University of Edinburgh, Edinburgh EH9 3FF, United Kingdom

**Keywords:** GPP, Eddy covariance, Droughts, Water-carbon coupling, Causal analysis, Empirical dynamic modelling, Temperate peatlands

## Abstract

Despite only covering ~3 % of the land mass, peatlands store more carbon (C) per unit area than any other ecosystem. This is due to the discrepancy between C fixed by the plants (Gross primary productivity (GPP)) and decomposition. However, this C is vulnerable to frequent, severe droughts and changes in the peatland microclimate. Plants play a vital role in ecosystem C dynamics under drought by mediating water loss to the atmosphere (surface water vapour conductance) and GPP by the presence/absence of stomatal regulation. This is dependent on soil moisture, air temperature, and vapour pressure deficit (VPD). Although there is ample evidence of the role of VPD on stomatal regulation and GPP, the impact of soil moisture is still debated. We addressed this knowledge gap by investigating the role of bulk surface conductance of water vapour in shifts between climatic (Air temperature (Tair), incoming shortwave radiation (SWR) and VPD) and water limitation of GPP in a peat bog in Canada. A causal analysis process was used to investigate how environmental factors influenced GPP. The results suggested that stomatal regulation in response to increased VPD caused the reduction in GPP in 2016 (~2.5 gC m^−2^ day^−1^ as opposed to ~3 gC m^−2^ day^−1^ in 2018). In contrast, GPP was limited again in 2019 due to the dry surface. This was driven by the relaxed stomatal regulation adopted by the ecosystem following the initial drought to maximise C assimilation. We found the threshold at which surface water decline limited GPP was at about −8 cm water table depth (82.5 % soil moisture). The causal inference corroborated our findings. The temporal variations of water and energy limitation seen in this study could increasingly restrict GPP due to the projected climate warming.

## Highlights

• The peatland preferred water retention over carbon assimilation.
• During the initial drought, the ecosystem responded with stomatal regulation to prevent water loss.
• Post drought the ecosystem implemented relaxed stomatal regulation to maximise carbon assimilation.
• The resulting high rates of evapotranspiration pushed the water table depth below – 8 cm and soil moisture below 82.5 %.
• GPP was limited by soil moisture as the water table depth dropped below this threshold.

## Graphical abstract

**Figure:**
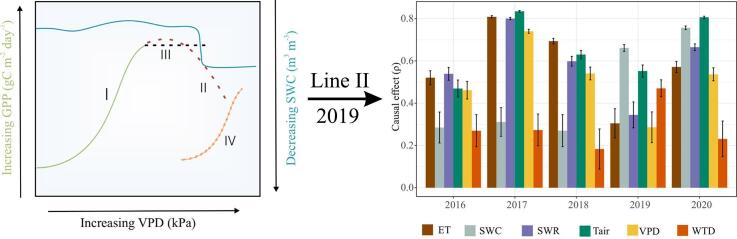

## 1 Introduction

Globally, peatlands store ~650 gigatons of carbon (GtC) (~20–25 % of the world's soil C). This massive C store is possible because of very slow decomposition of organic matter due to anoxic conditions (Kleinen et al., 2012; Nichols and Peteet, 2019; Turetsky et al., 2015; Yu, 2011). In their natural state, peatlands account for ~20 % of global CH_4_ emissions (Bloom et al., 2010) but the climatic effect of CH_4_ emissions can be outweighed by net CO_2_ uptake and storage over longer time scales (Frolking et al., 2011). Gross primary productivity (GPP) is of paramount importance in C accumulation and appraisal of terrestrial C dynamics (Grossiord et al., 2020; Kwon et al., 2022; López et al., 2021; Novick et al., 2016, Novick et al., 2024; Peichl et al., 2018; Perez-Quezada et al., 2024).

**Fig. 1:**
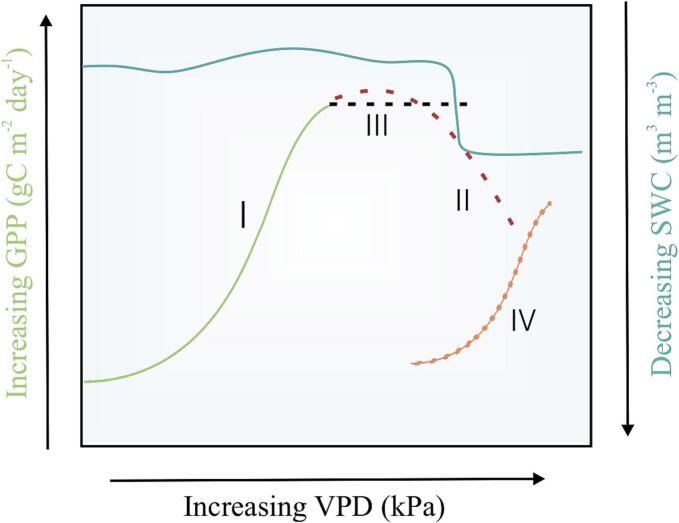
A conceptual diagram portraying ecosystem GPP responses to changes in VPD and SWC. Here four probable GPP responses (marked in Roman numerals Ι through IV) to changes in VPD and SWC are shown. I (Green line): GPP increases with an increase in VPD under normal VPD and SWC. II (Red dotted line): Partial or complete stomatal closure to preserve water leads to a sudden decline in GPP. III (Black dotted line): Under increased VPD (increased atmospheric water demand), ecosystems tend to increase iWUE to maximise C assimilation. This results in a constant GPP value. IV (Orange dotted line): With frequent droughts, ecosystem chooses an alternate steady state where GPP-VPD relationship mimics I. But photosynthetic efficiency (unit C fixed per unit leaf area) is reduced compared to I.

Plant water-C coupling and its drivers play a critical role in regulating GPP. There are two crucial points of water-C coupling: in the roots and the stomata (Gentine et al., 2019). The stomata are where atmospheric CO_2_ enters the leaf for carboxylation and, in the process, transpires water back into the atmosphere. Stomata essentially regulate the balance between water uptake from the soil, water loss through the leaves, and CO₂ intake, working in coordination with the roots (Liu et al., 2020; Sabot et al., 2022). These processes are heavily dependent on the climate, species, ecosystem state and vice-versa (Bassiouni et al., 2020; Grossiord et al., 2020; López et al., 2021; Zarakas et al., 2020). Peatland C - water exchanges are further complicated by the presence of *Sphagnum* moss, which lack stomata, and instead have passive capillary action for water transport and water retention characteristics (Kim and Verma, 1996; Nichols and Brown, 1980; Price, 1997; Thompson and Waddington, 2008; Kettridge and Waddington, 2014). But climate warming and increases in severity of extreme events threatens to alter plant water-C coupling and GPP in peatlands (Freeman et al., 2022; Frolking et al., 2011; Turetsky et al., 2015). Climate change is causing the severity and frequency of droughts to increase in the northern hemisphere, where most peatlands are located (IPCC, 2023). Droughts can also cause or combine with fire, resulting in a cascade of damage responses (Tschumi et al., 2022). For instance, severe atmospheric droughts trigger soil water content (SWC) decline due to increased atmospheric water demand (i.e., vapour pressure deficit (VPD)), exposing peat soils to oxygen and releasing CO_2_ (Fenner and Freeman, 2011). An increase in VPD has also been shown to reduce GPP under severe droughts because vascular plants close their stomata to prevent further water loss (Fig. 1; Line II. A change from linear positive relationship (Line I)) (Breshears et al., 2013). Thus, the ecosystem fails to meet the atmospheric water demand, and in time, this will cause a decrease in surface conductance of water vapour ($G_{\mathrm{sw}}$) (Grossiord et al., 2020; Novick et al., 2016). Plants can also increase their intrinsic water use efficiency (i.e. the ratio of C gained to water lost through stomata) by stomatal regulation to maximise C assimilation (Fig. 1; Line III) under increased VPD (Zhang et al., 2016). However, the role of SWC on GPP is still debated. While some studies suggested at the presence of SWC limitation (Wang et al., 2022; Voigt et al., 2024), and Zhang et al. (2016) showed the absence of SWC limitation. In peatlands, mosses complicate the SWC-GPP dynamics due to their lack of stomata. Consequently, mosses cannot prevent water loss (Admiral and Lafleur, 2007; Williams and Flanagan, 1996), resulting in desiccated and compressed mosses under droughts (Dorrepaal et al., 2004; Nichols and Brown, 1980; Thompson and Waddington, 2008). This, combined with soil texture properties, prevents soil evaporation because of a decline in capillary rise, causing the hysteresis between SWC and evapotranspiration (ET), culminating in lagged responses of GPP and stomatal or canopy regulations to drought. As the water table depth (WTD) drops, the peat and ecosystem properties change with prolonged droughts. These changes can push the ecosystem to an alternate steady state (Fig. 1 Line IV), such as shrub dominance which can also support C accumulation in peatlands (Wang et al., 2015).

Despite extensive literature covering broader terrestrial ecosystem GPP-VPD relationships and, more recently, SWC relationships, a consensus has not yet been reached on the VPD-SWC-GPP dynamics and the role of $G_{\mathrm{sw}}$ especially in drought-affected peatlands. To this extent, we ask the following research questions and respective scientific hypotheses to address the abovementioned knowledge gaps.

1. How does atmospheric drought impact $G_{\mathrm{sw}}$, ET and GPP in a restored peatland (Burns Bog) in Vancouver, Canada?
  We hypothesise that $G_{\mathrm{sw}}$ started declining with increasing drought severity due to physiological regulation leading to plant-water stress (Fig. 1; Line II).
2. Is there a threshold at which SWC drawback starts to regulate GPP?
  We hypothesise that variation in SWC beyond a point causes GPP to be regulated by SWC more than VPD.

## 2 Materials and methods

To address out research questions we carried out an ecosystem scale diagnostic analysis first to investigate the physiological and aerodynamic variability of water transport in drought-affected peatland using a bigleaf approach. Secondly, an approach called empirical dynamic modelling (EDM) (Sugihara et al., 2012) was used to estimate the consequent causal variability of environmental controls on GPP. The EDM was used to retrieve the time-varying causal links between GPP and its meteorological drivers in Burns bog, Vancouver, Canada. The model was driven by leveraging the publicly available eddy covariance (EC) dataset (CA-DBB; Christen and Knox, 2022) and in-situ observations from the site at weekly time step for five years from 2016 to 2020.

### 2.1 Description of the study site

The CA-DBB EC station is located in Burns Bog in Delta, British Columbia, Canada (49.129°N, −122.986°W). Burns Bog is a degraded ombrotrophic peat bog in the Fraser River Delta. Peat harvesting in the bog occurred between 1930 and 1970. The bog has since been reduced to approximately 58 % of its original size through drainage, harvesting, and encroachment of agricultural and industrial lands (Hebda et al., 2000). The bog was formally protected with the establishment of the Burns Bog Ecological Conservancy Area in 2001. Later, in 2007, rewetting via ditch blocking began to raise the water table and promote peat development.

The climate of Burns Bog is characterised by warm, dry summers and mild, wet winters (Köppen-Geiger climate classification scheme; Csb (Kottek et al., 2006)). Climate normal (1990–2020) from nearby Vancouver International Airport indicates the area receives peak precipitation between November and January (>170 mm/month), while drier conditions are experienced in July and August (<40 mm/month). In the winter, water flows radially from the centre to the edges of the bog, while in the summer, there is minimal lateral flow. Vegetation in Burns Bog is diverse; the area around the CA-DBB EC station is dominated by *Sphagnum* spp. moss and white beak sedge (*Rhynchospora alba*). More details on the study site are found in Lee et al. (2017) and Satriawan et al. (2023).

### 2.2 Data

#### 2.2.1 Eddy covariance data

We used publicly available EC data covering a five year period (2016–2020, CA-DBB; (Christen and Knox, 2022)). Carbon dioxide fluxes (F_C_) were calculated from 20 Hz EC data using EddyPro (Fratini and Mauder, 2014). The QA/QC filtering was done using the flagging system of Foken et al. (2004). Post-processing of F_C_ was done using ReddyProc (Wutzler et al., 2018). We used friction velocity ($u_{*}$) filtering and storage flux correction (S_C_) following Papale et al. (2006), to calculate Net Ecosystem Exchange (NEE) as NEE = F_C_ + S_C_. The night-time NEE partitioning approach (Reichstein et al., 2005) was used to estimate its component fluxes, ecosystem respiration (Reco) and GPP. These data were then aggregated to a weekly time step. The first week of 2016 was omitted because GPP and Reco were absent. Thus, the analysis included 259 weeks of EC data.

#### 2.2.2 In-situ data

We also used gap-filled estimates of SWC and WTD corrected for the seasonally variable height of the bog surface. SWC was measured half-hourly using a CS616 water content reflectometer. The probe was moved to a hummock in winter to avoid complete submersion. The probes were again moved to a hollow to avoid underestimating SWC when the hummock dried out in the summer months. Additionally, wildlife-associated disturbances necessitated the replacement of the original CS616 probe in the summer of 2019. To compensate for these inconsistencies, a combination of probe/position (four in total) multiple linear regression models trained to estimate SWC using rolling averages of air temperature, relative humidity and rolling sums of cumulative precipitation observed at the site over hourly, daily, and monthly (30-day) intervals. These four estimates were then averaged to calculate the SWC across the site and aggregated into weekly intervals. We caution that the values of SWC shown here may not represent absolute SWC at the site. Still, they are suitable for providing a relative estimate of SWC over the study period.

Water table depth was measured continuously over the study period using a pressure transducer inserted into a PVC pipe. The PVC pipe was fixed to a piece of rebar inserted deep into the sub-surface. This was done to prevent vertical drift in the position of the pressure transducer. A phenomenon known as “bog breathing” results in significant seasonal fluctuations of the elevation of the peat surface, up to 30 cm between annual maxima in mid-winter and minima in early fall. Manual measurements of water table depth and the height of the top of the PVC pipe above the bog surface were collected on routine maintenance visits. These observations were used to correct for sensor drift and account for the bog breathing phenomena. Furthermore, the measurements were used to scale the continuous measurements of water table height (relative to an arbitrary datum) to the actual water table depth relative to the bog surface.

### 2.3 Methods

#### 2.3.1 Detection of atmospheric dryness

We used a combination of the standard precipitation evapotranspiration index (SPEI) (Beguería et al., 2014; Vicente-Serrano et al., 2010a, Vicente-Serrano et al., 2010b, Vicente-Serrano et al., 2012, Vicente-Serrano et al., 2013) and moisture coefficient (Pereira et al., 2015; Allen et al., 1998) as an indicator of drought in the peatland. First, we estimated the moisture coefficient in the peatland. It is the ratio of ET to potential evapotranspiration (PET; Eq. (1)). Plants were considered water stressed in weeks with a value below 0.6.

(1) Moisture coefficient=ET/PET

PET is the water that would have evaporated and transpired if the ecosystem had enough water. PET was estimated using the Penman method (Penman, 1948) (Eq. (2)). The equation calculates daily potential evapotranspiration, combining an energy balance equation based on net radiation with an aerodynamic approach. We used the *R* programming environment (R 4.1.3) package Evapotranspiration (function ET.Penman) for modelling PET.

(2) $$\mathrm{PET}=\frac{s}{s+\gamma }.\frac{R_{n}}{\lambda }+\frac{\lambda }{s+\lambda }.E_{a}$$

where daily PET (in mm day^−1^) is from a saturated surface (aggregated by weeks for compatibility with the rest of the data), $R_{n}$ is the daily net shortwave radiation to the evaporating surface (in MJ m^−2^ day^−1^), s is the slope of the saturation vapour pressure curve (kPa °C^−1^) at a given air temperature, γ is the psychrometric constant (kPa °C^−1^), and λ is the latent heat of vaporisation (in MJ kg^−1^). $E_{a}$ (Eq. (3), in mm day^−1^) is a function of the average daily wind speed (u, in m s^−1^) and vapour pressure deficit (D, in kPa). It accounts for the aerodynamic component of the Penman equation. i.e. it includes the evapotranspiration driven by wind and vapour pressure deficit.

(3) $$E_{a}=f\left(u\right)D=f\left(u\right)\left(v_{a}^{*}-v_{a}\right)$$

where $v_{a}^{*}$ is the saturation vapour pressure (kPa). $v_{a}$ is the actual vapour pressure (kPa). $f\left(u\right)$ (Eq. (4)) is a function of wind speed (Penman, 1956):

(4) $$f\left(u\right)=2.626+1.381u$$

Next, we calculated SPEI using the *R* programming environment (R 4.1.3) package SPEI (version 1.8.1). SPEI compares the highest PET to current water availability. See Beguería et al. (2014), Vicente-Serrano et al. (2010a), and Vicente-Serrano et al. (2010b) for more details.

#### 2.3.2 Modelling the sensitivity of bulk surface conductance of water vapour to climatic stress

The surface conductance ($G_{\mathrm{sw}}$ (m s^−1^), Eq. (5)) to latent heat transfer was estimated by inverting the Penman-Monteith model for evapotranspiration (Knauer et al., 2018). Here, the vegetation is represented as a single uniform layer/one large canopy (i.e., ‘big leaf’). The model estimates the combined conductance of the soil and plant surface. $G_{\mathrm{sw}}$ was estimated at weekly timestep using the EC data.

(5) $$G_{\mathrm{sw}}=\frac{\mathrm{\lambda E}G_{\mathrm{ah}}\gamma }{s\left(H+\mathrm{\lambda E}\right)+\rho c_{p}G_{\mathrm{ah}}D-\mathrm{\lambda E}\left(s+\gamma \right)}$$

where $G_{\mathrm{ah}}$ is the bulk aerodynamic conductance for heat transfer (m s^−1^) (Eq. (6)), E is the evaporation rate or flux of water vapour, λE is the latent heat flux (W m^−2^), γ is the psychrometric constant (kPa K^−1^), s is the slope of the saturation vapour pressure curve (kPa K^−1^), Available energy (W m^−2^) was approximated using the sum of sensible heat flux (H) and λE (Humphreys et al., 2006).

(6) $$G_{\mathrm{ah}}={\left(R_{\mathrm{am}}+R_{\mathrm{bh}}\right)}^{-1}$$

$R_{\mathrm{am}}$ is the aerodynamic resistance to momentum transfer (or 1/$G_{\mathrm{am}}$, where $G_{\mathrm{am}}$ is the aerodynamic conductance; Eq. (8)) with turbulence as the principal transport mechanism. $R_{\mathrm{bh}}$ is the canopy (quasi-laminar) boundary layer resistance (“excess resistance”) to heat transfer (or 1/$G_{\mathrm{bh}}$, where $G_{\mathrm{bh}}$ is the quasi-laminar boundary layer conductance) (Massman, 1999; Verma, 1989.). We calculated $G_{\mathrm{bh}}$ as a function (Eq. (7)) of friction velocity ($u_{*}$, m s^−1^) following Thom (1972).

(7) $$G_{\mathrm{bh}}={\left(6.2{u_{*}}^{-0.67}\right)}^{-1}$$

$G_{\mathrm{am}}$ can be calculated as a function of $u_{*}$ and wind speed (m s^−1^) as seen below (Eq. (9)).

(8) $$G_{\mathrm{am}}=\frac{{u_{*}}^{2}}{u\left(z_{r}\right)}$$

Parameters in Eq. (6) to Eq. (8) can be estimated using the aerodynamic conductance function in the R package bigleaf (Knauer et al., 2018). The surface conductance function was used to calculate $G_{\mathrm{sw}}$ (Eq. (5)) at weekly timestep using $G_{\mathrm{ah}}$ estimate from above. These estimates were used to track intra-annual changes in $G_{\mathrm{sw}}$ in the drought-affected peatland across the five-year study period.

Finally, we estimated evaporative fraction (EF) as the ratio of latent heat flux (λE) and the sum of latent and sensible heat flux (λE +H) (Eq. (9)). This was to see how much of the available energy was used for evapotranspiration from the peat surface. We then plotted its relationship with SWC and $G_{\mathrm{sw}}$.

(9) $$\mathrm{EF}=\frac{\mathrm{\lambda E}}{\mathrm{\lambda E}+H}$$

#### 2.3.3 Empirical dynamic modelling (EDM)

EDM is an equation-free mechanistic model framework (Deyle et al., 2016; Deyle and Sugihara, 2011; Sugihara et al., 2012; Ye et al., 2015) centred around state space reconstruction (Deyle and Sugihara, 2011; Kugiumtzis, 1996; Vlachos and Kugiumtzis, 2009). It applies to complex dynamic systems where the relationships between the interacting variables vary with changes in the system state (Sugihara et al., 2012; Ye et al., 2015) and cannot be described by equations and the hypotheses and assumptions that come with it. EDM is thus only constrained by the amount and the quality of the data. The purpose of using EDM in this study was twofold: (a) to study the interactions between variables and (b) to detect causation. These two methods are described in detail below.

a. S-maps for measuring interactions between variables for each week

The ecosystem state is a specific point at a particular time in the multivariate coordinate axis (each axis would be the variables such as GPP, Tair, ET, SWC, WTD, and atmospheric CO_2_ concentration). These variables are causally coupled. The causality of these variables will change as time evolves, and changes in the magnitude of the variables themselves will lead to a shift in the ecosystem state. As such, interactions between the multiple variables must be recalculated sequentially for each ecosystem state. This can be done using S-maps (R package rEDM (Version: 1.14.0; function: smaps) (Deyle et al., 2016; Sugihara et al., 1996, Sugihara et al., 2012; Sugihara, 1994). The changes in interactions as the ecosystem state varied were used in the redundancy analysis to see how sensitive GPP, Reco, and the causal interactions were to the changes in the variables, as outlined below.

b. Convergent cross mapping (CCM) for retrieving causality

We used CCM to extract causation from the time series data (R package rEDM; Version: 1.14.0; function: ccm). At the heart of the CCM is its ability to detect and separate causation from spurious correlations in time series data of complex coupled dynamic systems. The analysis is based on the theory put forward by Takens (1981), which states that in a multidimensional dynamical system with only a finite number of observable variables, the essential information of the system is retained in the time series of any observed single variable of that system. This means we can replicate the system dynamics by using the lagged time series of the observable variable in place of the unknowns. Thus, if causation exists between two variables, it can be extracted from the time series history of the affected variable (cross-mapping). The effectiveness is then reported using the correlation coefficient (ρ,crossmapskill).

We examined the variation in causality between GPP and factors such as Tair, ET, VPD, atmospheric CO_2_ concentration, SWR, SWC, and WTD across the five-year study period (259 weeks together) and for each year with a time lag of 1 week by applying CCM (Sugihara et al., 2012). The cross-map skill, ρ, of each pair (GPP:ET, GPP:Tair and so on) was reported. The standard error (SE) of ρ was estimated by the equation below (Eq. (10)) (Gnambs, 2023).

(10) $$\mathrm{SE}=\frac{1-\rho^{2}}{\sqrt{N-3}}$$

where, N is the number of weeks (library size) in each step of the CCM.

#### 2.3.4 Redundancy analysis

Redundancy analysis (RDA) using the five-year data was performed (R package: vegan version 2.6-4; Oksanen et al., 2013) to extract the direction and magnitude (length of the arrows) of causal strength estimates from above, along with SWC, WTD, Tair, VPD, ET, SWR, and atmospheric CO_2_ concentration.

#### 2.3.5 Relative variable importance

A random forest regression was performed (R package: randomforest) on the factors used in the RDA, and then relative variable importance (relative to the most significant variable for estimating GPP) was calculated for predicting GPP. The adjusted R^2^ and corresponding *p* values of the random forest regression were reported.

#### 2.3.6 Cross correlation test

Additionally, we performed cross correlation between ET and SWC using the weekly estimates. The cross correlation test was used to assess the lag between ET and decrease in SWC. We reported correlation coefficient of the lag in weeks at 95 % confidence interval and the significance was assessed based on the p value.

**Fig. 2:**
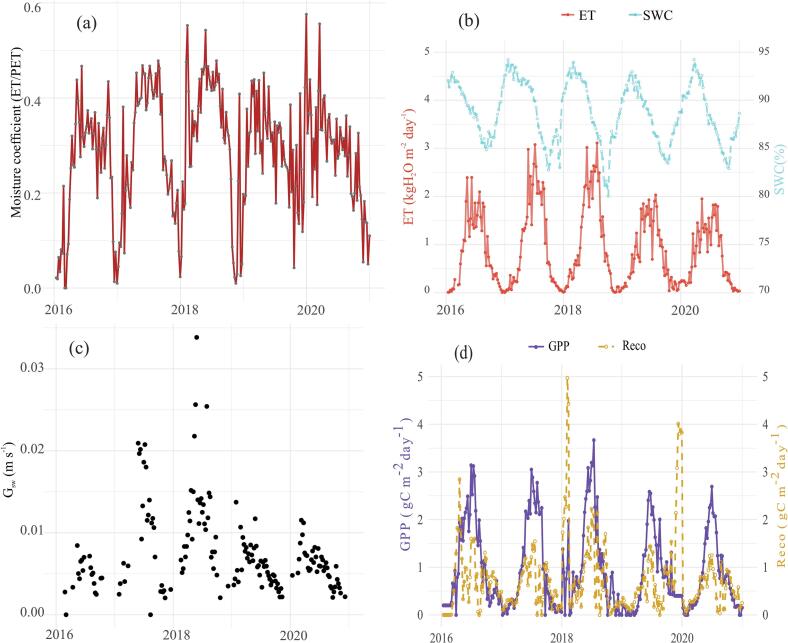
Weekly timestep water and carbon dynamics at Burns Bog for five years from 2016 to 2020. (a): The moisture coefficient shows the variability in the severity of the atmospheric drought. (b): temporal variability in SWC (blue dashed lines) and ET (orange lines). (c): intra-annual variability in $G_{\mathrm{sw}}$, and finally (d): variability in GPP (purple lines) and Reco (orange dashed lines).

All the analysis were carried out using the R programming language version 4.4.1 (R Core Team, 2024) in RStudio version 2024.04.2.764 (RStudio Team, 2024). The figures (excluding Fig. 2b) were made similarly. Fig. 2b was created using the TeX programming language (Knuth, 1986) in Overleaf (https://www.overleaf.com).

## 3 Results

### 3.1 Temporal variability of plant physiological controls under drought

Burns bog preferred to minimize water loss over C assimilation under the persistent atmospheric drought experienced (Fig. 2a, Fig. S1) during our study period (2016 to 2020). The moisture coefficient stayed below 0.5, indicating a weak plant-atmosphere coupling and plant-water stress. Whilst the coefficient was consistently below 0.4 in 2016 and 2019 (Fig. 2a and Table 1; higher plant-water stress), values around 0.5 were observed for 2017, 2018 and 2020 (Fig. 2a and Table 1; lower plant-water stress). We also observed an SPEI of ~−1.5 during the summer periods; in this, 2016 and 2020 were relatively severe (Table 1 and Fig. S1). The data reveal an asynchrony between drought severity (Fig. S1), plant-water stress, $G_{\mathrm{sw}}$ and GPP (Fig. 1 and Table 1). We looked at the SWC and ET time series data to determine the reasons for this ecosystem response.

**Table 1:** Annual variability of drought-related parameters. We considered the following criteria to classify the parameters into high and low years at Burns bog. The plant-water stress is considered relatively high if the fraction is below 0.4. Similarly, a drought is considered severe when SPEI is ≤−1.5. $G_{\mathrm{sw}}$ and ET were classified as high for years with $G_{\mathrm{sw}}$ ≥ 0.2 m s^−1^ and ET ≥ 3 kgH_2_O m^−2^ day^−1^. Decrease in SWC is considered high when it falls below 82.5 %. EF is considered high when it is above 0.5 (Fig. S3).

	Unit	High (years)	Low (years)
Plant-water stress	Fraction	2016, 2019	2017, 2018, 2020
Drought	Fraction	2016, 2020	2017, 2018, 2019
$G_{\mathrm{sw}}$	m s^−1^	2017, 2018	2016, 2019, 2020
ET	kgH_2_O m^−2^ day^−1^	2017, 2018	2019, 2020
SWC	%	2017, 2018	2016, 2019, 2020
EF	Fraction	2016, 2017, 2018	2019, 2020

In 2016, the drought was severe (Table 1, Fig. S1). Nevertheless, the ecosystem did not transpire a proportionate amount of water due to stomatal regulation (Table 1); low rates of ET and increased EF in 2016 imply high soil evaporation rates and low transpiration rates. In subsequent years (2017 and 2018), a reduction in plant-water stress – evidenced by high bulk surface conductance ($G_{\mathrm{sw}}$) and elevated EF, indicating minimal stomatal regulation (Fig. 2a, Table 1) – was attributed to decreased drought severity (Table 1, Fig. S1). This reduction in stress led to higher ET rates, reaching ~3 kg H₂O m^−2^ day^−1^ (Fig. 2b, Table 1). GPP was thus the highest in 2018 (~3.2 gC m^−2^ day^−1^).

The increase in ET caused the depletion of surface moisture, with SWC dropping to ~82.5 % in the latter part of 2017 and 80 % near the end of 2018 (Fig. 1b, Table 1). This coincided with a water table drawdown to −8 cm (Figs. S7, S9). VPD showed no notable differences during this period (Fig. S6). However, this SWC decline had a 14-week lagged response to ET (correlation coefficient of 0.77 (*p* < 0.001) at a 95 % confidence interval and 257 degrees of freedom, c.f., Fig. 2b and Fig. S12). This can be due to the hydraulic resistance properties of the peat soil (texture) and water availability (WTD was on or above the peat surface except for the drought severe months; Fig. S7).

Subsequently, in 2019, the EF dropped (Fig. S4) and the ecosystem experienced plant water stress again. This time due to the decline in SWC to below 82.5 % (and WTD of 8 cm or more below the peat surface, Fig. S7). The impact of SWC passing this threshold resulted in the decline of G_sw_ and rates of ET of ~1.8 gC m^−2^ day^−1^ (Fig. 2c, Table 1). Peak GPP of 2.5 gC m^−2^ day^−1^ was observed during this period (Fig. 2d). Interestingly, SWC dropped only to ~85 % during the non-growing season of 2019, similarly to the other years (Fig. 2b). Finally, 2020 recorded a severe drought year (Table 1, Fig. S1) and saw a similar GPP, SWC, and ET as 2016. However, the plant-water stress was not as high as in 2019. Also, while GPP generally exceeded Reco during the study period (Fig. 2, Figs. S1, S8) (Satriawan et al., 2023), the site became a net source of C to the atmosphere (11.9 ± 15.1 gC m^−2^ year^−1^) by 2020 (Satriawan et al., 2023).

### 3.2 Bidirectional causation strength across five years

**Fig. 3:**
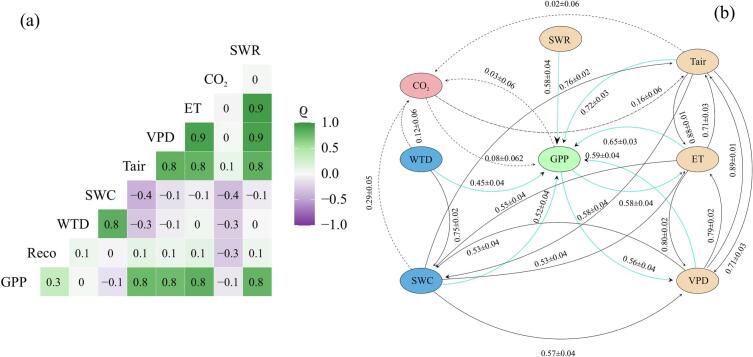
Five year (a): correlation matrix using the Pearson correlation coefficient. (b): multivariate causation cross-map skills retrieved using the CCM analysis. The cross-map skill is displayed using the Pearson correlation coefficient (ρ). The black dotted lines indicate weaker causal relationships. The coloured lines show the direct causal relationships between GPP and SWR, VPD, Tair, SWC, WTD, ET, or atmospheric CO_2_ concentration (indicated as CO_2_). The black solid lines indicate causation between variables.

Overall, energy and atmospheric related factors (VPD, Tair and SWR) regulated GPP across the five-year period (Fig. 3). GPP had strong climate-carbon feedback with both ET and VPD. The effect of ET on GPP (ET:GPP) was stronger (0.65 ± 0.03) than the impact of VPD (VPD:GPP; 0.59 ± 0.04) and SWR (VPD:SWR; 0.58 ± 0.04) on GPP. The effect of GPP (feedback) on these variables (GPP:ET and GPP:VPD) was equally strong (0.58 ± 0.04 and 0.52 ± 0.04, respectively). CCM revealed a significant impact of SWC and WTD on GPP (Fig. 3b). This was also evident from the temporal variability of plant physiological properties (Figs. 2c & S3). On the other hand, these findings differ from the correlation analysis (Fig. 3a). We also found that the impact of SWC on VPD (SWC:VPD) (0.57 ± 0.04) was greater than its effect on GPP (SWC:GPP; 0.52 ± 0.04, Fig. 3b). However, this was weaker than the effect of ET:GPP (0.65 ± 0.03), VPD:GPP (0.59 ± 0.04) and Tair:GPP (0.72 ± 0.03). ET and Tair exhibited a strong causal link (ET:Tair: 0.88 ± 0.01 and Tair:ET: 0.71 ± 0.03) and so did ET and VPD (ET:VPD: 0.80 ± 0.02 and VPD:ET: 0.79 ± 0.02). However, climate-carbon feedbacks between atmospheric CO_2_ concentration and GPP were insignificant in this five year dataset. This is because the CO_2_ concentrations did not increase over the period.

### 3.3 Temporal variability of energy and water limitation of GPP

**Fig. 4:**
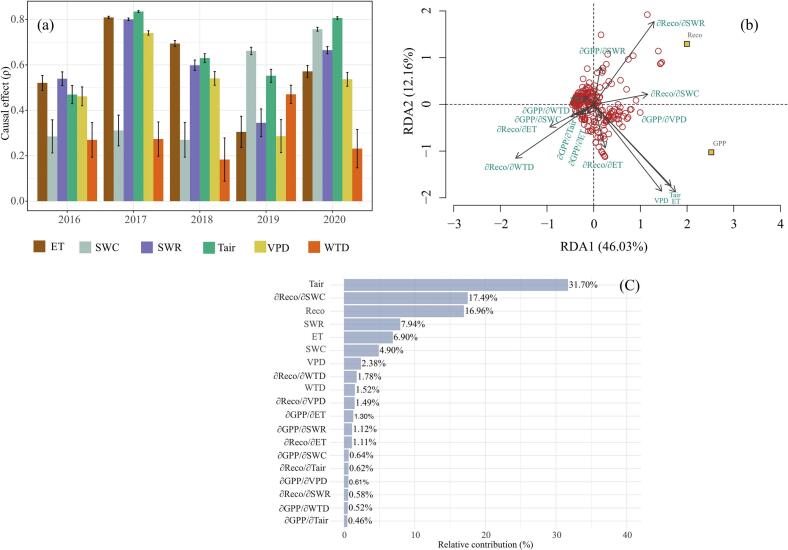
(a): Cross-map skill at convergence retrieved using the CCM analysis. Each of the bars represent the effect of the predictors on GPP (ET:GPP, VPD:GPP, etc.) for each year. Missing bars represent non-significant (ρ=0) effects. (b): RDA analysis showing the impact of causal interactions on GPP and Reco at a weekly timestep. (c): relative variable importance of causal interactions, carbon and energy-related parameters on GPP. The relative variable importance was extracted from random forest regression with 500 trees, with the model expanding 66 % of the variance in GPP with a root-mean-square error of 0.55 gC m^−2^ day^−1^.

The causal inference analysis revealed an interannual transition from energy limitation to water limitation for GPP (Fig. 4a). This is consistent with the physiological control and the decline in soil moisture (Section 3.1). CCM analysis on the weekly time series for each year revealed that in 2016, 2017 and 2018 GPP was energy limited. This can be seen from the stronger causal effect of VPD, ET, SWR and Tair on GPP relative to the influence of SWC and WTD (Fig. 4a). This corroborates the observed stomatal regulation and increased ET (2016, 2017 and 2018) against the severe drought of 2016. Energy-related factors significantly influenced GPP in 2017 and 2018, as plants prioritized carbon assimilation, resulting in ET compared to other years. However, in 2019, the causal effect of SWC:GPP was the highest and energy-related drivers had little to no causal effect on GPP. The shift from a VPD to water limitation of GPP is due to the decrease in SWC in 2018 and the lagged response of SWC to ET (as discussed above). In 2020, GPP was again driven by VPD and other energy related factors. This is because SWC did not drop below 82.5 % in 2019.

Next, we did a detailed exploration of weekly time step causal interaction strength variability (Section 2.3.3 b; S-maps) of GPP (δGPP) and Reco (δReco) with the same set of variables and its impact on both Reco and GPP (Fig. 4b). RDA (Section 2.3.4) was used to analyse the variability in the weekly timestep causal interactions. We found that along with changes in VPD, Tair and ET, the causal strength of Tair on Reco (δReco/δTair), SWC on Reco (δReco/δSWC), VPD on GPP (δGPP/δVPD) all had positive correlations with GPP. But the weekly causal variability of SWC on GPP (δGPP/δSWC), WTD on GPP (δGPP/δWTD), SWR on GPP (δGPP/δSWR), ET on Reco (δReco/δET) and SWC itself had a negative correlation with GPP. It again suggests that the ecosystem preferred water retention over C assimilation. This is also evident by the relative contributions these drivers had on the variability of GPP (Fig. 4c). Meanwhile, causal strength of ET on GPP (δGPP/δET), Tair on GPP (δGPP/δTair), Reco on WTD (δReco/δWTD), and ET on Reco (δReco/δET) had a positive correlation with GPP. However, these factors have strong negative correlations with Reco.

## 4 Discussion

Spatial and temporal dynamics of energy and water limitation of terrestrial GPP are central to understanding ecosystem CO_2_ dynamics and related uncertainties in dry and drought-affected zones (Ahmad et al., 2024; Fu et al., 2022; Heijmans et al., 2004; Kim and Verma, 1996; Schwingshackl et al., 2017; Seneviratne et al., 2010). Due to their status as the largest reservoirs of soil organic C, peatlands could have dire climate-carbon feedback depending on their sensitivity at varied timescales to droughts and warmer climates. In this study, we focused on the ecosystem physiological regulations of water vapour fluxes and finally displayed how, when, and what regulates GPP.

### 4.1 Temporal dynamics of physiological regulation of water vapour

Peatlands generally evaporate much like intact broadleaf forests in the temperate and boreal regions, but less than open-water evaporation (Baldocchi et al., 2000; Humphreys et al., 2006). This leads to a weak but significant energy limitation. Although WTD, SWC, and their relations with energy and C fluxes have been rigorously studied, their role in ET and photosynthetic changes is still not straightforward (Clymo and Hayward, 1982; Mezbahuddin et al., 2016; Moore et al., 2013; Nichols and Brown, 1980; Thompson and Waddington, 2008; Wu et al., 2010).

We show that in the first three years of the study, GPP was not limited by soil moisture (Fig. 2b, Table 1); instead it was influenced by the presence or absence of physiological control exerted by vascular plants to mitigate drought stress, driven by changes in Tair and VPD, a finding strongly supported in the literature (Novick et al., 2016; Grossiord et al., 2020; Ficklin and Novick., 2017). The isohydric nature led to the plant-water stress, contradicting the findings of canopy regulation in non-forested ecosystems to droughts (Zhang et al., 2016). This means that peatlands prefer to minimize water loss over C assimilation (Fig. 1 Line II). This might be why GPP was around 2.5 gC m^−2^ day^−1^ during the drought severe periods (Fig. 2. i.e. a decline in GPP is experienced as hypothesised in Fig. 1 line II). However, non-forested ecosystems included in Zhang et al. (2016) were grasslands and open shrublands. Access to copious amount of water and sphagnum dominance in comparison to shrublands or grasslands could be why the bog mimicked the forest ecosystem responses to drought as seen in Zhang et al. (2016). Our findings also differ from the diminished VPD suppression of vegetation growth in northern peatlands (Chen et al., 2023). The reduction/increase in the VPD suppression of GPP may be influenced soil moisture variability seen in this study. As the climate warms and droughts get more severe, the dual nature of GPP limitation as seen here could become more evident in the future. We think the decline in the severity of drought in the following years (2017, 2018) resulted in a relaxed physiological regulation to maximise C assimilation by the vascular plants (Chen et al., 2023). This led to higher ET and GPP, resulting in a decline in SWC, similar to Waddington et al. (2015).

After the decrease in SWC from the initial high rates of ET in 2017 and 2018, surface water started to limit both ET and GPP (Figs. 2b, d & 4a, Fig. S2) as observed in other studies (Satriawan et al., 2023; Humphreys et al., 2006). Up to this point, the EF was ≥0.5 (Figs. S3, S4, S5). This was reflected in $G_{\mathrm{sw}}$ (Fig. 2c), *G*_*ah*_ (Fig. S2), and thus in GPP (Fig. 2d). As a result, since 2018, especially from the start of 2019, EF declined (Fig. S5). Several laboratory and theoretical studies have documented the increase in moss surface water resistance with declining WTD (Nichols and Brown, 1980; Williams and Flanagan, 1996; Hayward and Clymo, 1982; Thompson and Waddington, 2008; Price et al., 2009; Kettridge and Waddington, 2014). This contributed to the hysteresis observed in the SWC-ET relationship, which exhibited a lagged response of 14 weeks (Fig. 2b, Fig. S12). Burns bog kept the water table above the surface in the summer months (Fig. S7; excluding 2016 and 2020), which meant there was enough water for ET. This could have led to the hysteresis. Although previous studies indicate that the threshold at which WTD limits ET is −40 to −50 cm (Kim and Verma, 1996; Mezbahuddin et al., 2015, Mezbahuddin et al., 2016), the WTD was −8 cm at our site when SWC started limiting GPP (Fig. S7). A recent study by Voigt et al. (2024) found that a WTD lower than −5 cm can limit GPP. As such, the SWC and WTD threshold for limiting ET and, thus, GPP may vary across peatlands, and limiting water levels may be lower than previously reported due to intensifying warming and rising VPD in the northern hemisphere (Helbig et al., 2020). Similar outcomes of energy balance and soil moisture control of ET have been reported for other terrestrial ecosystems (Bassiouni et al., 2020; Rodriguez-Iturbe, 2000; Schwingshackl et al., 2017).

Thus, we see combinations of GPP regulations at play; first, the physiological regulation (stomatal conductance) to changes in VPD and then a SWC limitation imposed due to a WTD decline as the result of much observed relaxed water restriction of peatlands after a severe drought.

### 4.2 Water and climatic controls on GPP

The causal process analysis further corroborated our finding that GPP was governed by SWC (Fig. 4a) after the decline in WTD to −8 cm and SWC to 82.5 % (Fig. S7; Fig. 2b). GPP was regulated by VPD (thus ET) and Tair in the first three years (Fig. 4a). After the drought-induced physiological regulation and an immediate relaxed stomatal regulation by the ecosystem led to water levels dropping to 8 cm below the surface, the ecosystem became water limited and GPP was reduced. The effect of SWC on GPP was higher in comparison with ET, VPD and Tair (Fig. 4a). The weekly timestep causal strength analysis (Fig. 4b) and the variable importance graph (Fig. 4c) support this point. These results are consistent with source-sink variability of NEE at Burns Bog, with the site being a net CO2 source of 11.9 ± 15.1 gC m^−2^ yr^−1^ in 2020 (Satriawan et al., 2023). Several studies provide evidence both supporting (Zhou et al., 2019a; Zhou et al., 2019b; Sulman et al., 2016) and opposing (Novick et al., 2016) the simultaneous limitation of GPP by VPD and soil moisture. Chen et al. (2023) report a reduction in the VPD suppression of GPP in the northern peatlands. This is significant considering the forecasts of intensifying climate warming and droughts, especially in the northern hemisphere (IPCC, 2023). In addition to ecosystem VPD limitation and suppression of GPP, a site scale atmospheric CO_2_ concentration manipulation (excess of 500 ppm) experiment showed a decline/desiccation of sphagnum cover in a temperate bog peatland (Norby et al., 2019). However, CO_2_ causal pathways (Fig. 3b) did not show any impact in this 5 year study, suggesting that temporal changes in VPD, Tair, ET, and SWC drove GPP over this period. The insignificant changes in CO_2_ concentration in Burns bog might be the primary reason for this. This is expected in the analysis of short period of time.

*Sphagnum* cover could have an important role to play in this. *Sphagnum* communities exhibit varying responses to frequent droughts and a warming climate. *Sphagnum fallax* species respond negatively to increasing droughts and temperatures, thus reducing canopy photosynthetic efficiency. On the contrary, *Sphagnum medium* tends to be drought-resistant (Jassey and Signarbieux, 2019). This nature of sphagnum communities can help the ecosystem maintain its functions even under reduced photosynthetic capacity. The presence of *Sphagnum* species has a role in modulating soil moisture supply for photosynthesis by mosses and vascular species present at the sites.

## 5 Conclusion

Our analysis showed that the rewetted bog preferred to minimize water loss over carbon assimilation under drought. We further identified a tipping point at which decreases in surface water start to regulate GPP. During the initial drought, the vascular plants regulated water loss through stomatal closure. Following this, the ecosystem relaxed the stomatal regulation to maximise carbon assimilation post drought. This led to a water table drawdown below the critical threshold. Dried out peat soil in combination with the water retention properties of the moss limited GPP. We identified this threshold in SWC to be 82.5 % (or a WTD of −8 cm). Following this decline in surface moisture, in the following year, soil moisture regulated GPP more than VPD, Tair, SWR or ET. Finally, even though WTD recovered above the threshold of −8 cm, the second severe drought put the ecosystem under stress again. However, we did not observe any plant water stress. Similar ecosystem responses are highly likely across northern peatlands in the future, driven by the projected increase in climate warming and the intensification of drought severity.

## CRediT authorship contribution statement

**Sandeep Thayamkottu:** Writing – review & editing, Writing – original draft, Visualization, Validation, Methodology, Investigation, Funding acquisition, Formal analysis, Data curation. **Mohit Masta:** Writing – review & editing, Writing – original draft. **June Skeeter:** Writing – review & editing, Validation, Resources, Methodology, Formal analysis, Data curation. **Jaan Pärn:** Writing – review & editing, Visualization, Validation, Supervision, Resources. **Sara H. Knox:** Writing – review & editing, Visualization, Validation, Resources, Methodology, Funding acquisition, Data curation. **T. Luke Smallman:** Writing – review & editing, Visualization, Validation, Supervision, Funding acquisition. **Ülo Mander:** Writing – review & editing, Visualization, Validation, Supervision, Resources.

## Declaration of competing interest

The authors declare that they have no known competing financial interests or personal relationships that could have appeared to influence the work reported in this paper.

## Data Availability

Eddy covariance data used in this study are available through Ameriflux: https://ameriflux.lbl.gov/at doi:10.17190/AMF/1881565. Data used in this study, outputs, and code to replicate the analysis and generate the figures are available through FigShare: https://figshare.com at https://doi.org/10.6084/m9.figshare.26485033.

## Appendix A Supplementary data

Supplementary figures Image 1
