# Supplementary material for: Dual controls of vapour pressure deficit and soil moisture on photosynthesis in a restored temperate bog
Source: Sci Total Environ. 2025 Feb 1;963:178366. doi: 10.1016/j.scitotenv.2024.178366 (PMC11772154; doi:10.1016/j.scitotenv.2024.178366)
Supplement: Supplementary file 1 — Supplementary figures [file mmc1.docx]

Supporting Information for

Dual controls of vapour pressure deficit and soil Moisture on photosynthesis in a restored temperate bog

S. Thayamkottu^a^, M. Masta^a^, J. Skeeter^b^, J. Pärn^a^, S.H. Knox^b,c^, T.L. Smallman^d,e^,

and Ü. Mander^a^

^a^Institute of Ecology and Earth Sciences, University of Tartu, Vanemuise Street. 46, 51003 Tartu, Estonia.

^b^Department of Geography, The University of British Columbia, Vancouver, BC, Canada.

^c^Department of Geography, McGill University, Montreal, QC, Canada

^d^School of GeoSciences, The University of Edinburgh, Edinburgh EH9 3FF, United Kingdom

^e^National Centre for Earth Observation, The University of Edinburgh, Edinburgh EH9 3FF, United Kingdom

This document contains supporting figures for the main text.


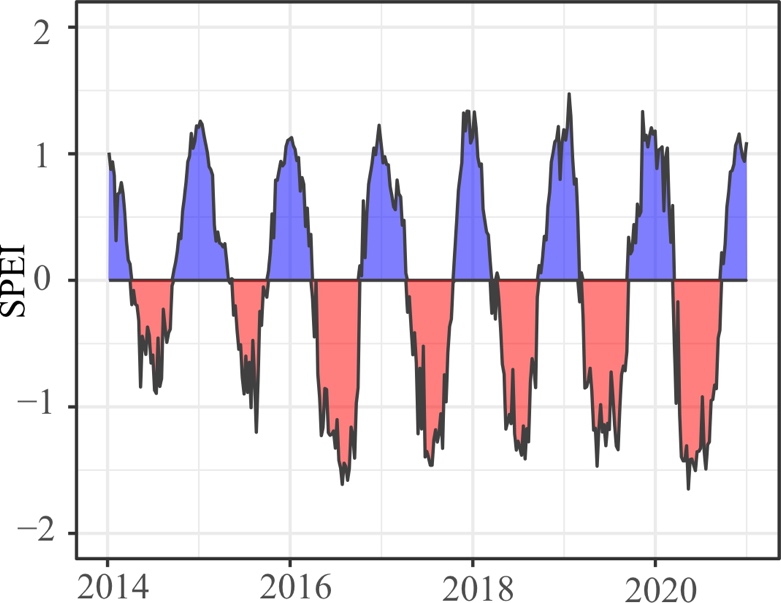


Figure S1. Weekly estimates of standard evapotranspiration precipitation index (SPEI). The negative values indicate a dry climate (red shaded region). The severity of atmospheric dryness increases as the SPEI values drop (peaks in summer), and the positive values (blue shaded region) indicate wet climate (peaks in winter).

**
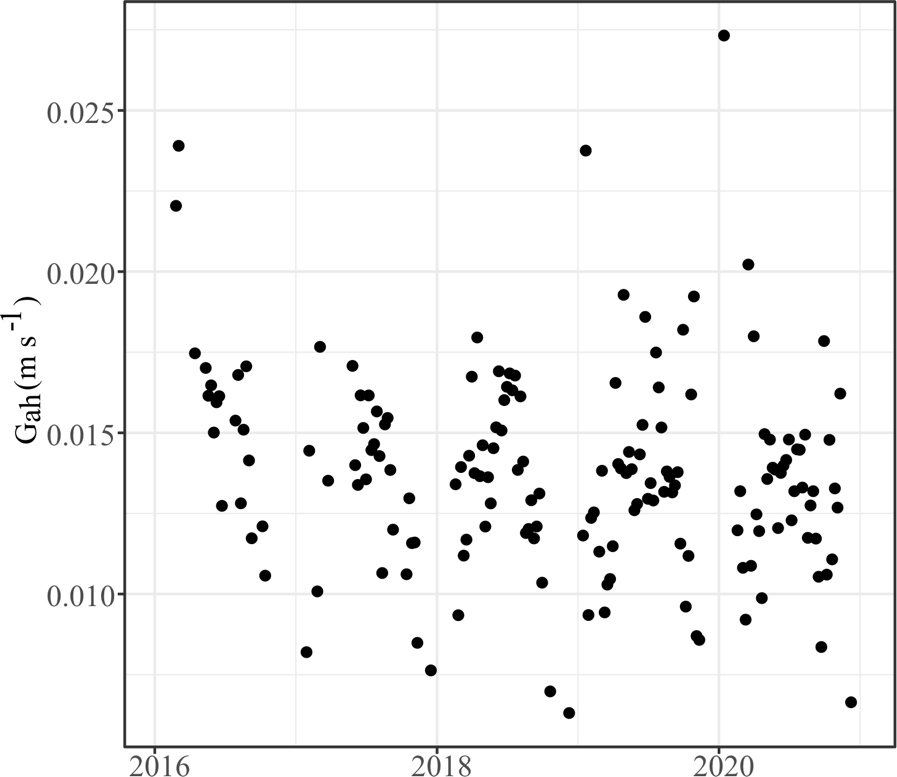
**

**Figure S2.** Bulk aerodynamic conductance of heat transfer ($G_{ah}$) estimated at a weekly time step. The aerodynamic variability was assessed using the big leaf approach (See section 2.3.2 in the main text for more details).

**
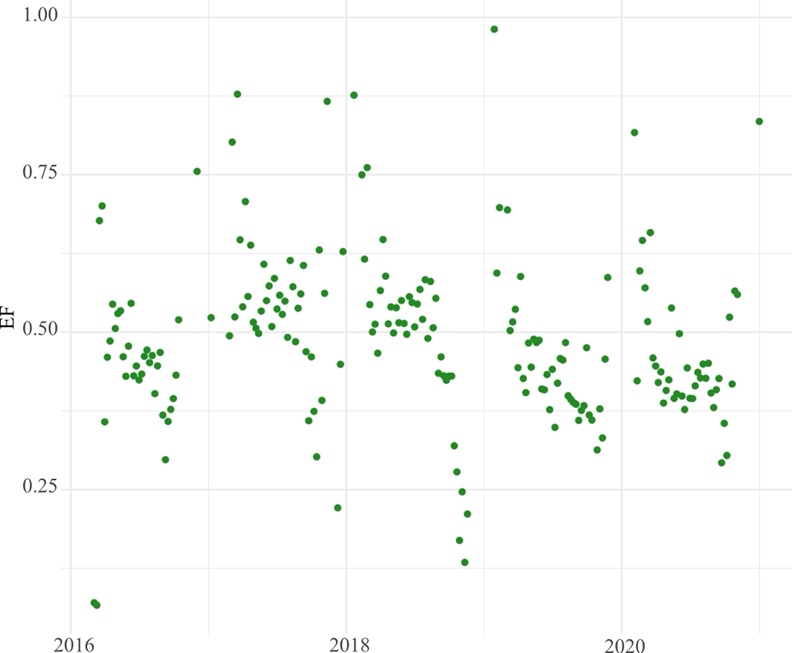
**

**Figure S3.** Weekly estimates of evaporative fraction (EF) from 2016 to 2020. The EF was the highest during the intense drought years of 2017 and 2018.

**
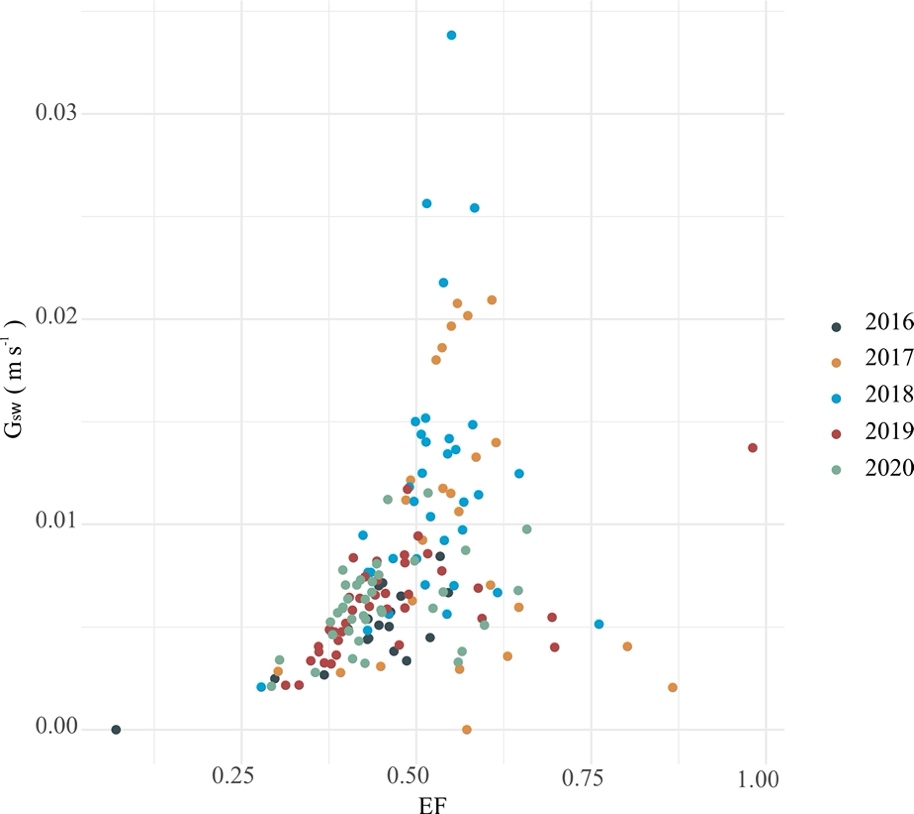
**

**Figure S4.** The relationship between weekly estimates of EF and $G_{sw}$. The colours represent each of the five years.


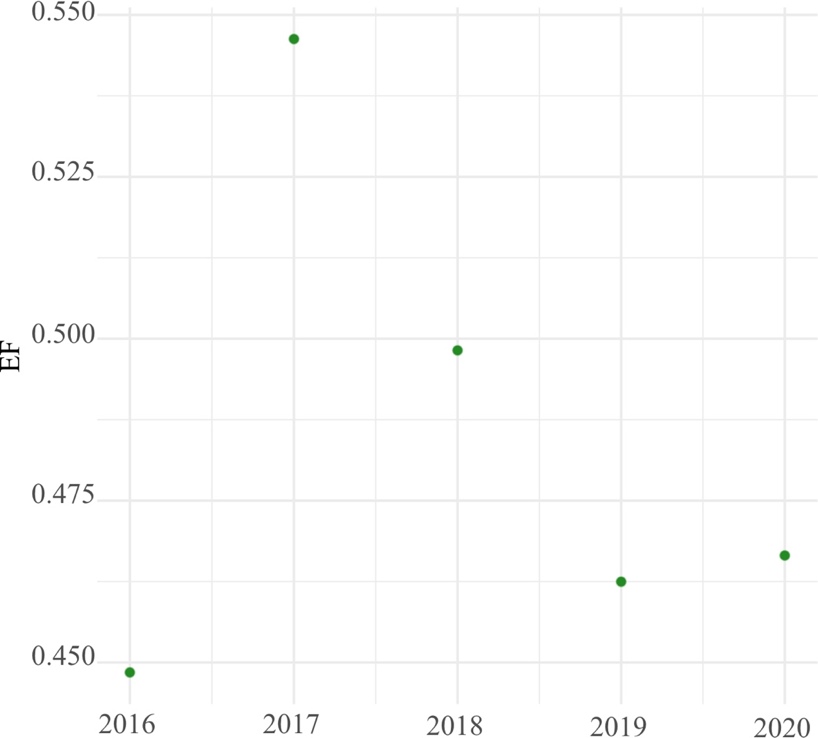


**Figure S5.** The site experienced a declining EF trend following 2017.

**Figure S6.** The time series of VPD at a weekly time step for five years.

**Figure S7**. The time series of WTD at a weekly time step. The missing values were gap-filled (See methods for more details).

**Figure S8**. The time series of NEE at a weekly time step for five years.


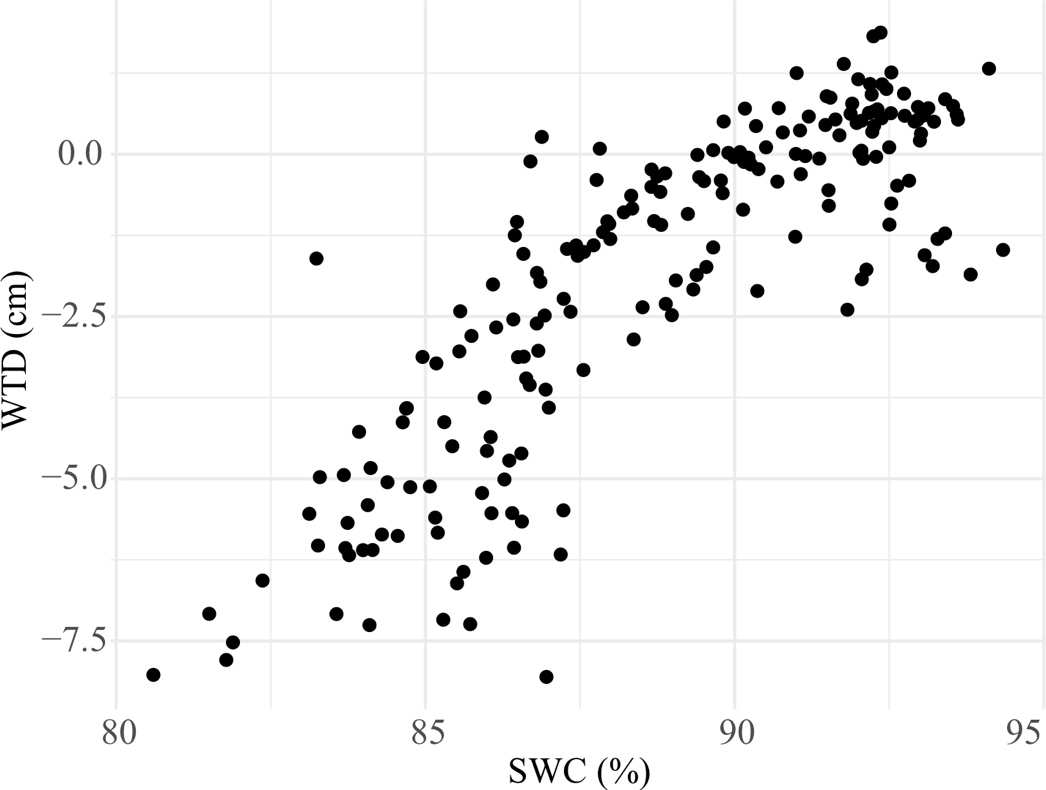


**Figure S9.** The linear trend between SWC and WTD


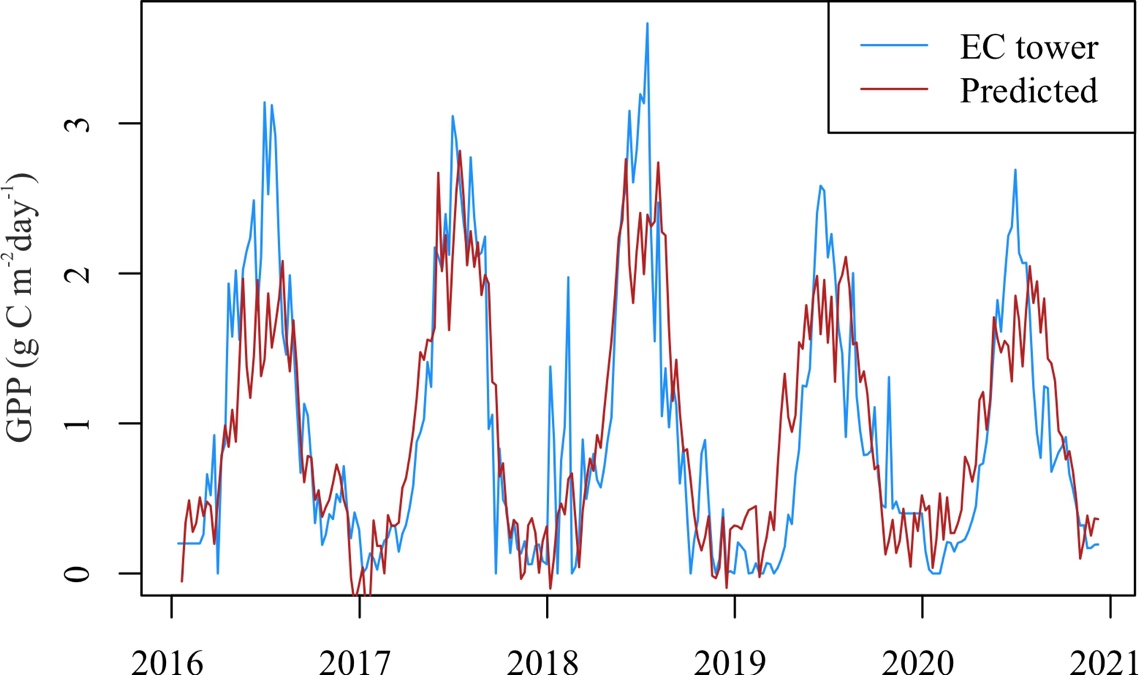


**Figure S10.** Time series of EDM retrieved and eddy covariance (EC) GPP.


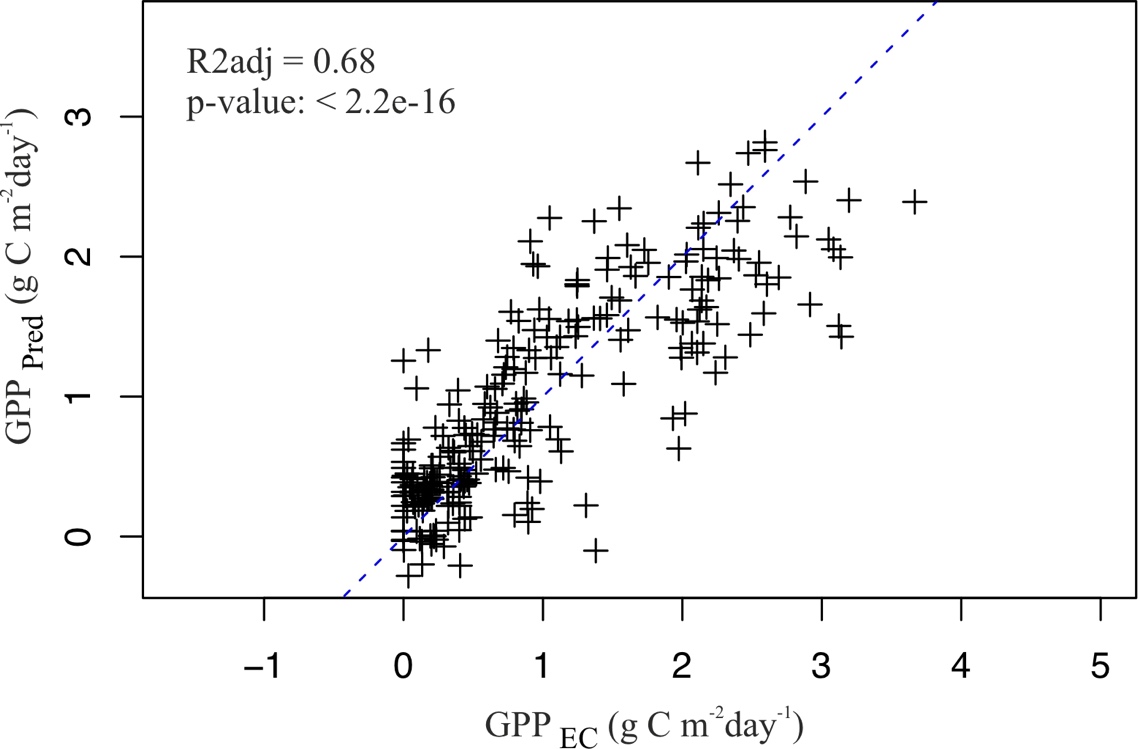


**Figure S11.** Scatter plot of eddy covariance (EC) and EDM retrieved GPP for a period of five years.


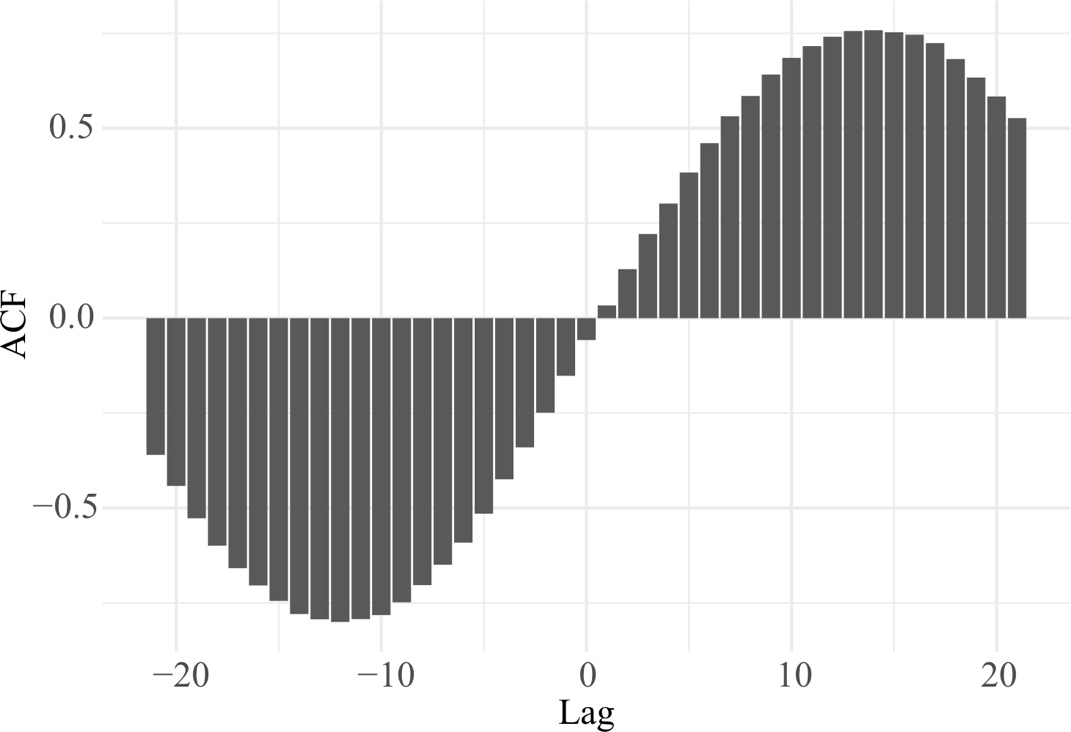


Figure S12: A sinusoidal plot of the lag between ET, SWC and their autocorrelation. A lag of ~14 weeks is seen in SWC response to ET. A negative lag and negative correlation (ACF) show a less evaporative demand indicative of the absence of drought. A positive lag suggests that changes in ET (high rates of ET) leads to a lagged SWC drawback
